# The adenoviral E1A N-terminal domain represses MYC transcription in human cancer cells by targeting both p300 and TRRAP and inhibiting MYC promoter acetylation of H3K18 and H4K16

**DOI:** 10.18632/genesandcancer.99

**Published:** 2016-03

**Authors:** Ling-Jun Zhao, Paul M. Loewenstein, Maurice Green

**Affiliations:** ^1^ Institute for Molecular Virology, Department of Microbiology and Molecular Immunology, Saint Louis University School of Medicine, Doisy Research Center, St. Louis, Missouri, USA

**Keywords:** adenoviral E1A 1-80, transcriptional repression, MYC, HER2, H3K18Ac, H4K16Ac, p300/CBP, TRRAP

## Abstract

Human cancers frequently arise from increased expression of proto-oncogenes, such as MYC and HER2. Understanding the cellular pathways regulating the transcription and expression of proto-oncogenes is important for targeted therapies for cancer treatment. Adenoviral (Ad) E1A 243R (243 aa residues) is a viral oncoprotein that interacts with key regulators of gene transcription and cell proliferation. We have shown previously that the 80 amino acid N-terminal transcriptional repression domain of E1A 243R (E1A 1-80) can target the histone acetyltransferase (HAT) p300 and repress HER2 in the HER2-overexpressing human breast cancer cell line SKBR3. Expression of E1A 1-80 induces death of SKBR3 and other cancer cell lines. In this study, we performed total cell RNA sequence analysis and identified MYC as the regulatory gene for cellular proliferation most strongly repressed by E1A 1-80. By RT-quantitative PCR analysis we show that repression of MYC in SKBR3 cells occurs early after expression of E1A 1-80, suggesting that MYC may be an early responder of E1A 1-80-mediated transcriptional repression. Of interest, while E1A 1-80 repression of MYC occurs in all eight human cancer cell lines examined, repression of HER2 is cell-type dependent. We demonstrate by ChIP analysis that MYC transcriptional repression by E1A 1-80 is associated with inhibition of acetylation of H3K18 and H4K16 on the MYC promoter, as well as inhibition of RNA Pol II binding to the MYC promoter. Deletion mutant analysis of E1A 1-80 suggests that both p300/CBP and TRRAP are involved in E1A 1-80 repression of MYC transcription. Further, E1A 1-80 interaction with p300/CBP and TRRAP is correlated with inhibition of H3K18 and H4K16 acetylation on the MYC promoter, respectively. Our results indicate that E1A 1-80 may target two important pathways for histone modification to repress transcription in human cancer cells.

## INTRODUCTION

Ad E1A 243R (243 amino acid residues) is a viral oncogene product that intimately interacts with cell cycle regulatory pathways to establish a favorable environment for viral DNA replication. E1A functions as a master regulator of chromatin remodeling and promoter activity by targeting the histone acetyltransferase (HAT) p300/CBP and TRRAP through the N-terminal region and conserved region 1 (CR1), tumor suppressor Rb through CR2, and transcriptional co-repressor CtBP through C-terminal CR4 (for a review, see [1]). E1A interaction with Rb is sufficient for inducing G1-S phase transition of cell cycle through activation of E2F. Of significance, E1A interaction with p300/CBP and CtBP has been shown to be anti-proliferative and this property has the potential to be harnessed for anti-cancer therapies [2, 3].

The N-terminal 80 aa residues of E1A 243R, E1A 1-80, encodes a transcriptional repression function, and induces the death of human cancer cells [3]. E1A 1-80 contains the N-terminal region and CR1, which together are sufficient for interaction of E1A 243R with p300/CBP and TRRAP [4-6]. p300/CBP plays critical roles in gene expression through histone acetylation, chromatin remodeling, and interactions with RNA Pol II and other transcription factors. TRRAP is a scaffold protein which assembles multi-subunit HAT-containing complexes for gene regulation [7]. We have shown that E1A 1-80 represses HER2 transcription in the human breast cancer cell line SKBR3 which over-expresses HER2 [3]. We proposed that breast cancer cells with HER2 over-expression may be HER2-addicted, and thus repression of HER2 expression by E1A 1-80 is a potential mechanism for E1A 1-80-mediated killing of these cells [3].

Subsequently, we reported that E1A 1-80 enhances p300 autoacetylation *in vitro*, and inhibits p300-mediated histone H3K18 acetylation *in vivo* and on reconstituted chromatin [8]. Since H3K18 hyper-acetylation is correlated with promoter activation [9], it is possible that E1A 1-80 inhibits H3K18 acetylation on the HER2 promoter, in part, to repress HER2 expression. Our studies *in vitro* have also shown that E1A 1-80 can dissociate TBP from a naked DNA promoter through interaction with p300 and TBP [6], suggesting that E1A 1-80 may use multiple mechanisms for transcriptional repression.

In this report, we identified by RNA-seq analysis the proto-oncogene MYC as the regulatory gene most strongly repressed by E1A 1-80. Both p300/CBP and TRRAP appear to be involved in E1A 1-80 repression of MYC. The MYC family genes are pivotal sensors of signal transduction pathways and regulators of cell proliferation, mostly by activation of gene transcription (for reviews, see [10, 11]). Nearly 50% of human cancers have increased MYC expression, and most human cancers require the function of MYC to survive, rendering MYC an attractive target for cancer therapy [12-14]. We show here that E1A 1-80 represses MYC in all eight human cancer cell lines examined, whereas HER2 repression by E1A 1-80 is cell-type-dependent.

## RESULTS

### HER2 repression by E1A 1-80 is cell-type dependent

**Figure 1:**
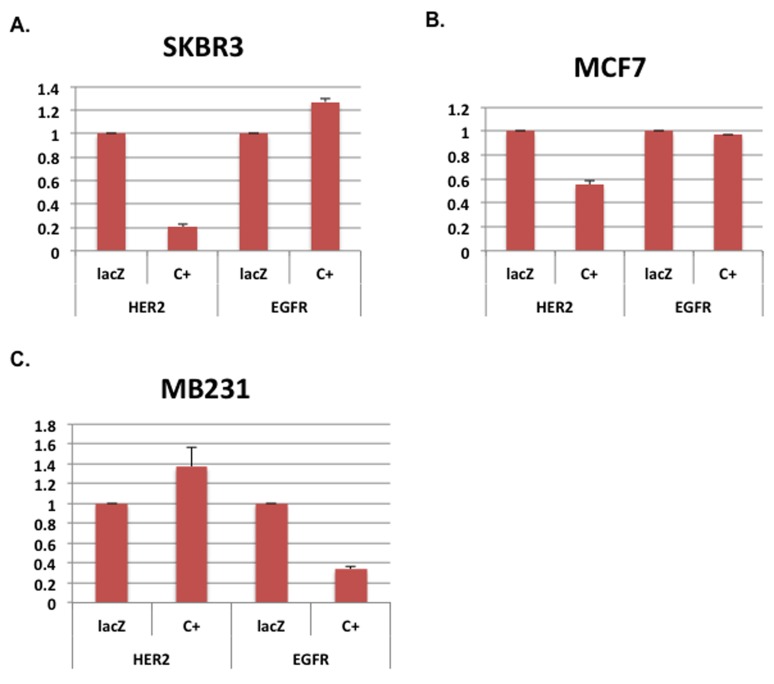
E1A 1-80 represses HER2 and EGFR in a cell-type dependent manner **A.** SKBR3 cells were infected with 20 PFU/cell of Ad-lacZ or Ad-E1A 1-80 C+ for 22 h, RNA was prepared and RT-qPCR performed with primers specific for HER2 and EGFR. Expression levels of HER2 and EGFR were normalized to levels in cells infected with Ad-lacZ, with GAPDH as an internal control. Data plotted are averages of two independent experiments with two batches of cells infected with Ad-lacZ (lacZ) or Ad-E1A 1-80 C+ (C+). Error bars are deviations from the averages. **B.**, **C.** MCF7 and MB231 cells, respectively, were analyzed as described for SKBR3 cells in A.

We have reported that SKBR3 cells, a human breast cancer cell line that over-expresses HER2, are efficiently killed by expression of E1A 1-80 [3]. Cell killing appears to correlate with E1A 1-80 repression of HER2 transcription in SKBR3 cells. In these studies, we expressed a modified E1A 1-80 with a C-terminal V5 tag from an Ad vector (Ad-E1A 1-80 C+) which expressed a higher level of E1A 1-80 and induced more efficient cancer cell death [3]. To compare E1A 1-80 regulation of HER2 and EGFR, another HER2 family member, SKBR3 cells were infected with Ad-E1A 1-80 C+ (expressed from the CMV promoter) or the control vector Ad-lacZ, and RT-quantitative PCR (RT-qPCR) analysis performed for EGFR and HER2. As shown in Figure 1A, E1A 1-80 C+ represses HER2 mRNA expression by ~ 80%, but does not repress EGFR mRNA expression.

To examine if E1A 1-80 regulates transcription of HER2 and EGFR in other breast cancer cell lines, MCF7 and MB231 cells were infected with Ad-lacZ or Ad-E1A 1-80 C+, and RT-qPCR analysis performed. E1A 1-80 C+ represses transcription of HER2 but not EGFR in MCF7 cells (Figure 1B), whereas in MB231 cells (Figure 1C), E1A 1-80 C+ inhibits EGFR and not HER2. From these data, it seems likely that E1A 1-80 regulation of HER2 and EGFR is cell-type dependent.

### Identification of MYC by RNA-seq analysis as a major target of E1A 1-80 transcriptional repression

To further understand transcriptional repression by E1A 1-80, we performed total cell RNA-seq analysis with triplicate RNA samples prepared from SKBR3 cells infected with Ad-lacZ or Ad-E1A 1-80 C+. RNA-seq analysis with oligo-dT-selected mRNA revealed a number of genes whose expression is modulated by E1A 1-80 C+ (see Supplementary Table 1). We selected six genes (Table 1) based on their strong repression by E1A 1-80 and their likely involvement in three broad categories of cellular regulatory functions: cell proliferation (MYC, Cyclin D1 (CCND1)), signal transduction (HER2, RHOB, TGFB3), and transcription (MYC, BCL3). As expected, HER2 was repressed efficiently by E1A 1-80 C+ (Table 1), consistent with the results of RT-qPCR analysis (Figure 1A).

**Table 1:** Selected regulatory genes strongly repressed by E1A 1-80 C+

		RNA-seq(1)	RT-qPCR(1)		
Gene Name	Description	SKBR3	SKBR3	MB231	MCF7
MYC	v-myc avian myelocytomatosis viral oncogene homolog	0.05	0.14	0.19	0.29
CCND1	cyclin D1	0.1	0.23	0.27	0.52
BCL3	B-cell CLL/lymphoma 3	0.14	0.17	0.26	0.54
ERBB2 (HER2)	erb-b2 receptor tyrosine kinase 2	0.12	0.2	1.39	0.56
RHOB	ras homolog family member B	0.1	0.23	0.89	0.35
TGFB3	transforming growth factor, beta 3	0.05	0.11	1.42	0.41

(1) Numbers are ratios of mRNA level in Ad-E1A 1-80 C+ infected cells / mRNA level in Ad-lacZ infected cells. RNA-seq results are averages from triplicate RNA samples, and RT-qPCR results are averages from duplicate RNA samples.

**Figure 2:**
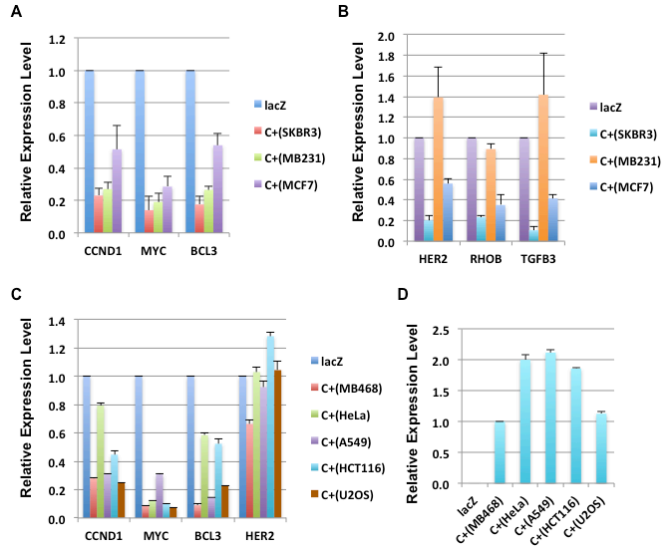
E1A 1-80 repression of genes identified by RNA-seq analysis **A.** Genes repressed by E1A 1-80 C+ in the three human breast cancer cell lines indicated. RT-qPCR was performed as described in Figure 1A. **B.** Genes efficiently repressed by E1A 1-80 C+ in SKBR3 cells and MCF7 cells, but not in MB231 cells. **C.** Repression of selected genes by E1A 1-80 C+ in the five human cancer cell lines indicated. **D.** Expression level of E1A 1-80 C+ in the cell lines used. Data are normalized to the level of E1A 1-80 in MB468 cells.

E1A 1-80 repression of the selected genes in SKBR3 cells was confirmed by RT-qPCR analysis, and further examined in two additional human breast cancer cells lines MB231 and MCF7 that are also efficiently killed by E1A 1-80 C+ [3]. As shown in Table 1 and Figure 2A, the genes most strongly repressed by E1A 1-80 C+ in all three cell lines are CCND1, MYC, and BCL3. Of interest, MYC is over-expressed in a large portion of human cancers and its function appears to be required for the majority of human cancers [12]. CCND1 is frequently amplified/overexpressed in human cancers [15], and BCL3 is a candidate proto-oncogene whose function is reportedly required for metastasis of HER2-positive breast cancer cells [16]. The other group of genes, including HER2, RHOB, and TGFB3 (transforming growth factor beta 3), displayed cell-type dependent regulation by E1A 1-80 C+ (Figure 2B): they were all repressed by E1A 1-80 C+ in SKBR3 and MCF7 cells, but not in MB231 cells.

Among the genes analyzed, MYC is the most strongly repressed by E1A 1-80 C+ in the breast cancer cell lines tested. To examine if MYC is efficiently repressed by E1A 1-80 C+ in cell lines derived from different cancer types, additional human cancer cell lines were infected with Ad-lacZ or Ad-E1A 1-80 C+, and RT-qPCR was performed. Among the cell lines chosen, MB468 is derived from a breast cancer, HeLa from a cervical cancer, A549 from a lung cancer, HCT116 from a colon cancer, and U2OS from an osteosarcoma. MYC is efficiently repressed by E1A 1-80 C+ in all cell lines tested (Figure 2C). BCL3 is also repressed efficiently in MB468, A549 and U2OS cells, whereas HER2 is repressed only in MB468 cells. E1A 1-80 C+ repression of CCND1 is also observed in all cell lines but generally at a lower level than MYC or BCL3. Examination of E1A 1-80 C+ transcripts shows comparable levels in the five cell lines (Figure 2D). Thus, these results confirm the general nature of MYC repression by E1A 1-80 C+ in all cancer cell lines tested (for a summary of RT-qPCR results, see Table 2A).

**Table 2:** Summary of RT-qPCR and ChIP-qPCR data

A. Ratio of level with E1A 1-80 C+ / level with lacZ (from Figure 2)						
	c-MYC	HER2				
SKBR3	0.14	0.20				
MB231	0.19	1.39				
MCF7	0.29	0.56				
MB468	0.09	0.66				
HeLa	0.12	1.03				
A549	0.31	0.92				
HCT116	0.1	1.28				
U2OS	0.07	1.05				
**B. Ratio of level with E1A 1-80 C+ / level with lacZ (from Figure 3)**						
			**Epigenetic modification**			
	**Pol II Binding**		**H3K18Ac**		**H4K16Ac**	
	**c-MYC**	**HER2**	**c-MYC**	**HER2**	**c-MYC**	**HER2**
**SKBR3**	0.13	0.31	0.18	0.35	0.38	0.99

### E1A 1-80 inhibition of H3K18 and H4K16 acetylation on the MYC promoter in SKBR3 cells

**Figure 3:**
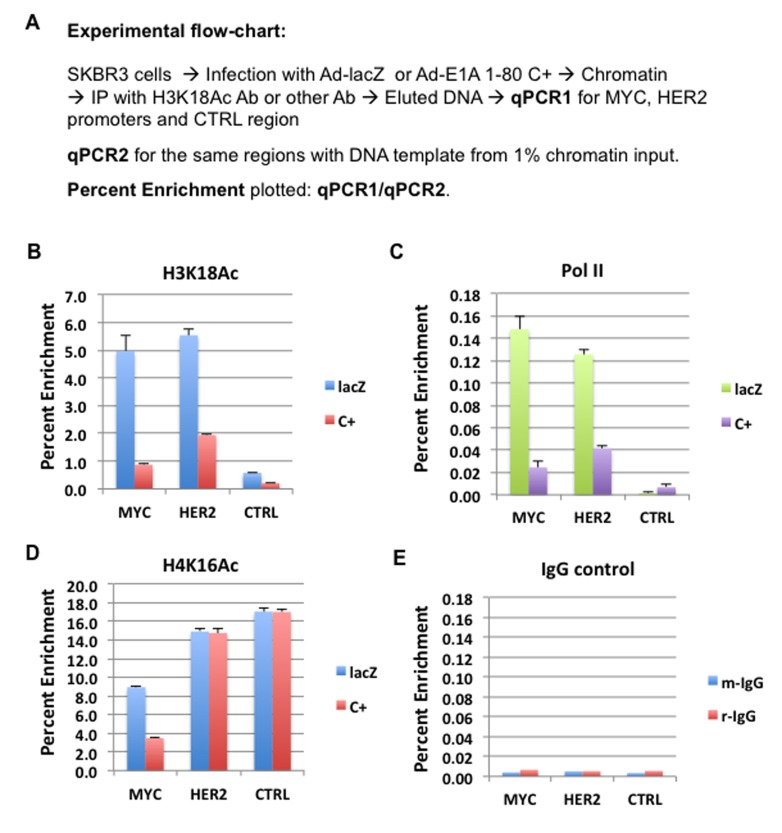
E1A 1-80 inhibition of RNA Pol II binding to and H3K18 acetylation on MYC and HER2 promoters in SKBR3 cells **A.** ChIP-qPCR experimental flow-chart. Percent enrichment of each promoter region is plotted. Percent enrichment is the ratio of qPCR1 from the ChIP DNA divided by qPCR2 from 1% input DNA. **B.** ChIP with H3K18Ac antibody. SKBR3 cells were infected with 20 PFU/cell of Ad-E1A 1-80 C+ or Ad-lacZ for 22 h, and fixed with formaldehyde before preparation of chromatin. CTRL: a control genomic region, ~ 4 kb upstream of HER2 transcription start site. MYC, HER2, and CTRL qPCR amounts were compared to the corresponding amounts from the “input” DNA. Each ChIP assay was repeated with the same chromatin preparation, and the averages of the two ChIP-qPCR assays are plotted, with error bars representing average deviations. **C.**, **D.** ChIP with RNA Pol II, and H4K16Ac antibodies, respectively. ChIP-qPCR was performed as in B. The different amounts of enrichment between the panels are likely due to different efficiencies of the antibodies used. **E.** ChIP control with non-specific antibodies, rabbit IgG (r-IgG) and mouse IgG (m-IgG).

It was previously reported that E1A 243R inhibits global H3K18 acetylation, an epigenetic mark catalyzed by p300/CBP [9]. We have shown that E1A 1-80 is sufficient to inhibit H3K18 acetylation *in vivo* and *in vitro* on assembled chromatin [8]. In addition, E1A 1-80 enhances p300 autoacetylation which appears to be correlated with the inhibition of H3K18 acetylation on chromatin. To examine the effects of E1A 1-80 on H3K18 acetylation on the MYC promoter *in vivo*, SKBR3 cells were infected with Ad-lacZ or Ad-E1A 1-80 C+, fixed with formaldehyde and processed for ChIP analysis as described in Figure 3A. ChIP DNAs were analyzed by qPCR for MYC and HER2 promoter regions, as well as a randomly selected control region ~ 4 kb upstream of the HER2 transcription start site (CTRL). Input DNA (1%) was directly analyzed by qPCR analysis with the same primers so that enrichment of target DNA regions by ChIP could be quantified. The H3K18Ac antibody enriched the MYC promoter by approximately 5% in cells infected with Ad-lacZ, but only 1% in cells infected with Ad-E1A 1-80 C+ (Figure 3B and Table 2B). Thus, H3K18 acetylation was repressed 80% by E1A 1-80 C+. H3K18 acetylation on the HER2 promoter was also repressed by E1A 1-80 C+. As expected, H3K18 acetylation on the CTRL region was low, and was inhibited to a lesser extent by E1A 1-80 C+.

Assembly of RNA Pol II on the promoter is a prerequisite for gene transcription. We therefore performed ChIP assay with Pol II antibody. Pol II antibody enriched MYC promoter by less than 0.2% (Figure 3C) in cells infected with Ad-lacZ. The lower enrichment compared to the H3K18Ac antibody is likely due to a lower antibody efficiency. Nevertheless, E1A 1-80 C+ efficiently reduces the Pol II occupancy on the MYC promoter. Pol II occupancy on the HER2 promoter is also reduced by E1A 1-80 C+, although with somewhat lower efficiently than on the MYC promoter. As expected, Pol II binding to the CTRL region was only marginal.

Acetylation of H4K16 is associated with transcriptionally active genes in mouse embryonic stem cells and is independent of p300/CBP activity [17]. H4K16 acetylation appears to be mediated by Tip60 or MOF [18-20], and is also involved in regulation of DNA damage repair [21]. To examine possible involvement of H4K16 acetylation in repression of MYC by E1A 1-80, ChIP assay was performed with antibody against H4K16Ac. As shown in Figure 3D, both the MYC and HER2 promoters, as well as the CTRL region, had high levels of H4K16 acetylation in Ad-lacZ infected control cells. However, E1A 1-80 C+ inhibits H4K16 acetylation on the MYC promoter but has only a minor effect on H4K16 acetylation on the HER2 promoter, suggesting that H4K16 acetylation plays a more important role for E1A 1-80 repression of MYC than for HER2.

As negative control, chromatin from cells infected with Ad-lacZ was immunoprecipitated with non-specific mouse or rabbit IgG, and the ChIP DNAs examined for the presence of MYC, HER2, and CTRL by qPCR (Figure 3E). As shown, both antibodies bound only marginal amounts of HER2 and MYC promoter regions as well as the CTRL region, showing that nonspecific binding of both mouse and rabbit IgG was low.

These combined results show that E1A 1-80 C+ strongly inhibits H3K18 acetylation and Pol II binding to the MYC promoter in SKBR3 cells. By comparison, E1A 1-80 C+ inhibits H3K18 acetylation and Pol II binding to the HER2 promoter to a lesser extent. However, E1A 1-80 C+ represses H4K16 acetylation only on the MYC promoter (see summary of ChIP assay data in Table 2B).

### MYC is an early target of transcriptional repression by E1A 1-80

**Figure 4:**
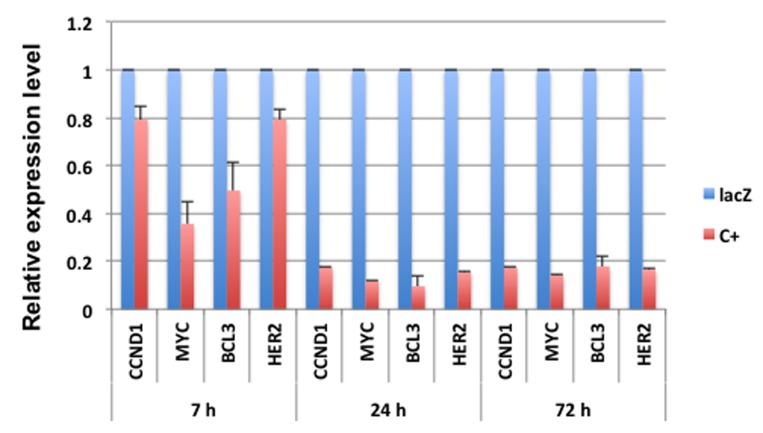
MYC is an early responder of transcriptional repression by E1A 1-80 Repression of selected genes by E1A 1-80 C+ at 7 h, 24 h and 72 h post infection. SKBR3 cells were infected in 6-well plates with 20 PFU/cell of Ad-lacZ (lacZ) or Ad-E1A 1-80 C+ (C+) and RT-qPCR conditions were as described in Figure 1.

Repression of cellular genes by E1A 1-80 may proceed in an orderly fashion, with some genes transcriptionally repressed earlier than other genes. It is possible that early responder genes are directly targeted by E1A 1-80. Since CCND1, MYC, BCL3 and HER2 are efficiently repressed by E1A 1-80 in SKBR3 cells (Figure 2A, 2B), we infected SKBR3 cells with Ad-lacZ or Ad-E1A 1-80 C+, and examined the mRNA levels of these genes by RT-qPCR at different times after Ad vector infection. As shown (Figure 4), efficient repression of MYC and BCL3 by E1A 1-80 occurred by 7 h after Ad vector infection, while repression of CCND1 and HER2 was not as efficient at this early time. Repression of all four genes was maximal by 24 h after infection, and this pattern persisted for at least 72 h after infection. These results suggest that MYC is an early responder to E1A 1-80 transcriptional repression, but repression of HER2 and CCND1 requires longer time of E1A 1-80 expression. However, once these genes are repressed by E1A 1-80, their expression levels do not appreciably change with time.

### E1A 1-80 repression of MYC involves p300/CBP and TRRAP

**Figure 5:**
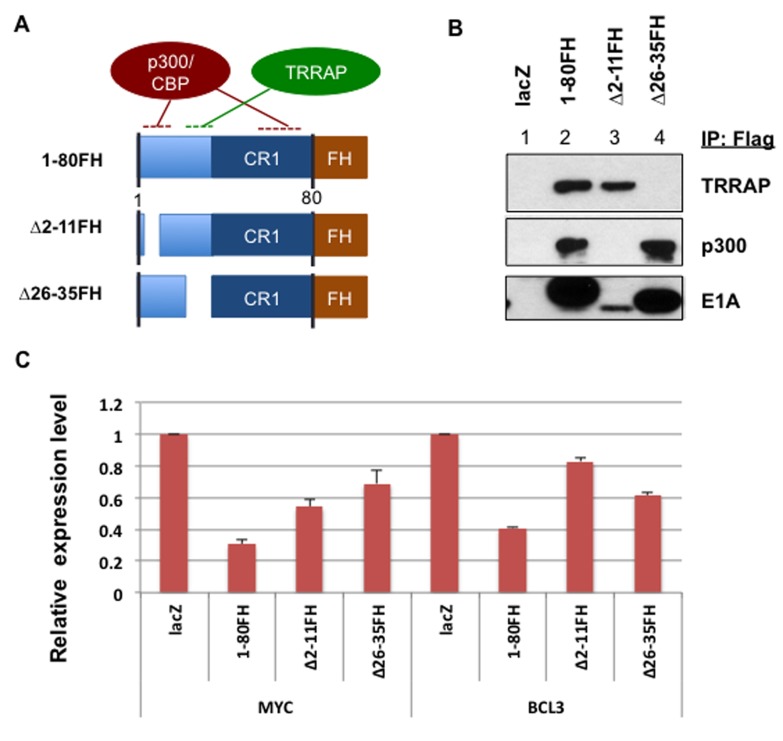
p300/CBP and TRRAP are involved in transcriptional repression of MYC by E1A 1-80 **A.** Illustration of E1A 1-80 domains and deletions potentially affecting interaction of E1A 1-80 with p300/CBP or TRRAP. FH: Flag-HA tag at the C-terminus of E1A 1-80. **B.** Interaction of E1A 1-80FH and deletion mutants with p300 and TRRAP. HeLa cells in T75 flasks were infected with 20 PFU/cell of Ad-E1A 1-80FH (lane 2), Ad-∆2-11FH (lane 3), and Ad-∆26-35FH (lane 4), harvested 22 h later for cell lysis, and cell extracts co-immunoprecipited with Flag-antibody beads. Bound proteins were eluted and examined by Western blot with the antibody indicated. E1A ∆2-11FH (lane 3) interacted with TRRAP well (top panel) but its Western blot level was apparently low (bottom panel), probably due to its small size and a low binding affinity to PVDF membrane. **C.** Effects of E1A 1-80FH and deletion mutants on transcriptional repression of MYC and BCL3. SKBR3 cells were infected with 10 PFU/cell of the indicated Ad vectors in 6-well plates for 22 h, and RT-qPCR performed. Data plotted represent average results from two independent RNA preparations, with error bars indicating deviations from the averages.

E1A 1-80 contains sequences that target p300/CBP and TRRAP. Interaction with p300/CBP requires two subdomains [22-24], and interaction with TRRAP requires a sequence immediately before conserved region 1 (CR1) (Figure 5A) [5]. To examine the requirements for transcriptional repression by E1A 1-80, we constructed Ad vectors expressing wild type E1A 1-80 with a Flag-HA tag on the C-terminus (E1A 1-80FH), and deletion mutants ∆2-11FH and ∆26-35FH, which in the context of E1A 243R weaken interaction with p300/CBP and TRRAP, respectively [4, 5].

The ability of these FH-tagged E1A 1-80 proteins to interact with p300 and TRRAP was examined by infection of HeLa cells with these Ad vectors, followed by immunoprecipitation of the cell lysates with Flag antibody beads and Western blot analysis with the indicated antibodies (Figure 5B). As shown, E1A 1-80FH co-precipitated both TRRAP (lane 2, top panel) and p300 (middle panel) as expected. ∆2-11FH did not interact with p300 (lane 3) but retained ability to interact with TRRAP. In contrast, ∆26-35FH interacted with p300 (lane 4) but not TRRAP.

To examine the roles of p300/CBP and TRRAP in E1A 1-80 mediated transcriptional repression, SKBR3 cells were infected with the control Ad-lacZ vector, or Ad vectors expressing E1A 1-80FH or its deletion mutants (Figure 5A). RT-qPCR was performed for MYC and BCL3, because both appear to be early responders to E1A 1-80 repression (Figure 4). As shown, E1A 1-80FH repressed both MYC and BCL3 efficiently (Figure 5C). Deletion of aa 2-11(∆2-11FH) or aa 26-35 (∆26-35FH) rendered E1A 1-80FH defective in repression of both genes. Importantly, ∆26-35FH was more defective than ∆2-11FH for repression of MYC, whereas the pattern was reversed for repression of BCL3. Thus, it appears that TRRAP, which is targeted by aa 26-35 of E1A 1-80, plays a more important role for repression of MYC, and p300, which is targeted in part by aa 2-11, is more important for repression of BCL3.

### Inhibition of H3K18 and H4K16 acetylation on the MYC promoter is correlated with E1A 1-80 targeting of 300/CBP and TRRAP, respectively

**Figure 6:**
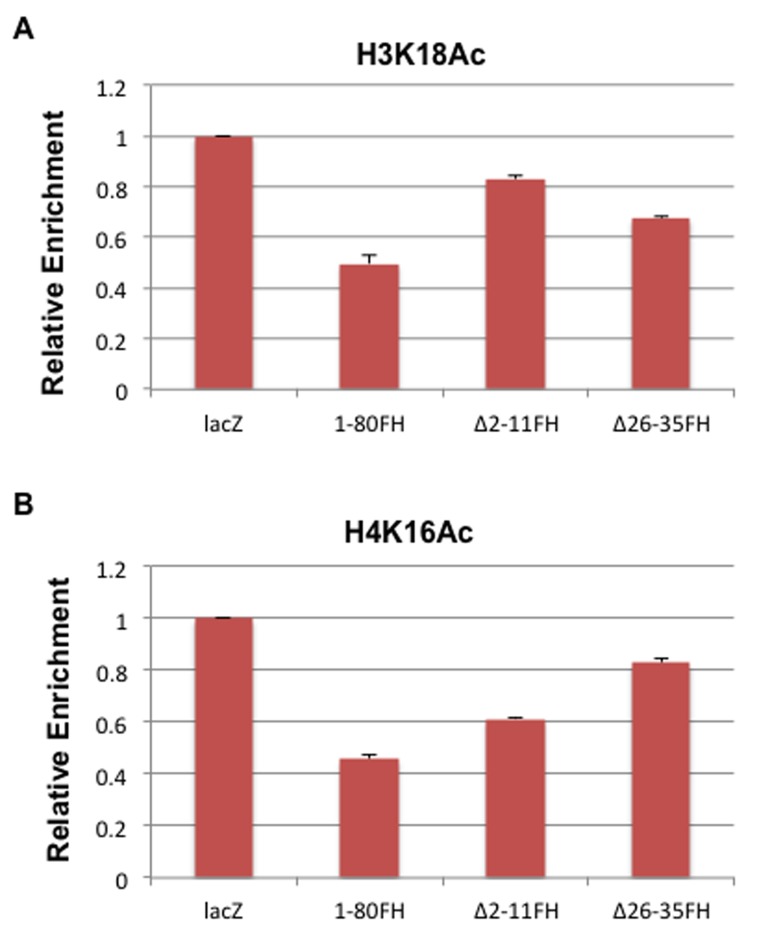
Inhibition of H3K18 and H4K16 acetylation is correlated with E1A 1-80 targeting of p300 and TRRAP, respectively SKBR3 cells were infected for 16 h with 10 PFU/cell of Ad-lacZ, or Ad vectors expressing wild type or the different deletion mutants of E1A 1-80FH. Chromatin was prepared for ChIP analysis with the H3K18Ac antibody in A, and with the H4K16Ac antibody in B. ChIP DNAs were analyzed by qPCR for the MYC promoter under the same conditions as in Figure 3. Each ChIP was done twice with the same preparations of chromatin, and the average data plotted with error bars indicating deviations from the averages. Percent enrichment of each ChIP assay was normalized to that of the cells infected with Ad-lacZ.

We previously demonstrated that E1A 1-80 inhibits p300-mediated H3K18 acetylation on *in vitro* assembled chromatin [8]. Results in Figure 3 show that H3K18 acetylation is also inhibited *in vivo* on MYC and HER2 promoters by E1A 1-80. Since the ∆2-11FH mutant of E1A 1-80FH does not target p300 and is partially defective in repression of MYC (Figure 5), we asked whether this mutant is also defective in inhibition of H3K18 acetylation on the MYC promoter. To examine this possibility, SKBR3 cells were infected with Ad-lacZ and Ad vectors expressing E1A 1-80FH and the two deletion mutants (Figure 5A), and ChIP assays were performed with antibody to H3K18Ac. As shown (Figure 6A), E1A 1-80FH inhibited H3K18 acetylation on the MYC promoter. As expected, the ∆2-11FH mutant was more defective than ∆26-35FH in this activity. Thus, p300/CBP targeting by E1A 1-80 appears to be required for inhibition of H3K18 acetylation on the MYC promoter.

Results described in Figures 3 show that acetylation of H4K16 on the MYC promoter was also inhibited by E1A 1-80. Since the ∆26-35FH mutant is defective in targeting TRRAP and also defective for repression of MYC (Figure 5), we performed ChIP assays with the H4K16Ac antibody using the chromatin preparations described above. TRRAP has been shown to be responsible for the assembly of multi-subunit HAT complexes, one of which contains Tip60 which favors acetylation of H4K16 [18]. As shown in Figure 6B, E1A 1-80FH inhibited H4K16 acetylation on the MYC promoter by approximately 50%. However, in contrast to the results with H3K18 acetylation (Figure 6A), the ∆26-35FH mutant was more defective than ∆2-11FH in inhibition of H4K16 acetylation. These results suggest that TRRAP function is important for E1A 1-80 to inhibit H4K16 acetylation on the MYC promoter.

## DISCUSSION

The adenovirus E1A N-terminal 80 amino acid domain is important for understanding transcriptional repression, cell transformation, and the regulation of cell proliferation. We previously identified HER2 as a gene efficiently repressed by E1A 1-80 in HER2 up-regulated SKBR3 cells [3]. Further, expression of E1A 1-80 resulted in death of SKBR3 cells as well as a number of other human cancer cell lines. In this report, MYC is identified by RNA-seq analysis as the regulatory gene most strongly repressed by E1A 1-80 in SKBR3 cells (Figure 2). Further, MYC is strongly repressed by E1A 1-80 in all eight cancer cell lines derived from different origins, whereas HER2 repression is cell line dependent (Figures 1 and 2, and Table 2A). The MYC proto-oncogene is over-expressed in a large portion of human cancers [12]. Knock-down of MYC in cancer cell lines can induce cell death [25-27]. MYC over-expression in solid human cancers often results from abnormal transcriptional or posttranslational regulation [12, 28-31]. Despite the discovery of many transcription factors that bind to the MYC promoter (for a review, see [31]), how the MYC promoter is regulated remains to be fully elucidated.

**Figure 7:**
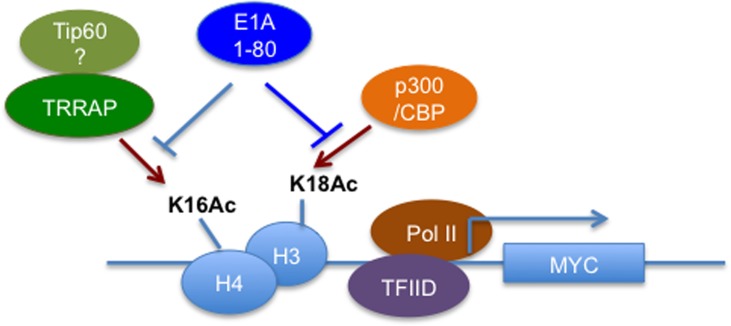
Potential model of transcriptional repression of the MYC promoter by E1A 1-80 E1A 1-80 targets p300/CBP and suppresses p300/CBP-mediated H3K18 acetylation. It also targets TRRAP, which associates with a HAT, for example Tip60, to inhibit H4K16 acetylation. Reduced acetylation of H3K18 and H4K16 may inhibit the recruitment of Pol II to the MYC promoter, contributing to the transcriptional repression of MYC.

In this report, we provide evidence that E1A 1-80 represses MYC transcription by targeting both p300/CBP and TRRAP pathways (Figure 7) and inhibiting the acetylation of H3K18 and H4K16 on the MYC promoter (Figure 3). Acetylation of H4K16 has been reported to be involved in transcriptional regulation of some promoters [17]. E1A 1-80 may directly inhibit HAT enzymes besides p300/CBP by targeting TRRAP (Figure 7), which is required for several HAT enzymes including, for example, Tip60 [18]. This could result in reduced H4K16 acetylation on the MYC promoter. This possibility is supported by the observation that ∆26-35FH is more defective than ∆2-11FH in the inhibition of H4K16 acetylation on the MYC promoter (Figure 6B) as well as being more defective in repression of MYC transcription (Figure 5C). Thus, TRRAP appears to play a more important role than p300/CBP in MYC repression by E1A 1-80. In contrast, E1A 1-80 repression of other genes, such as BCL3 (Figure 5C), appears to depend more on targeting p300/CBP.

In addition to inhibition of H4K16 acetylation on the MYC promoter, E1A 1-80 also inhibits H3K18 acetylation on both the MYC and HER2 promoters (Figure 3). p300/CBP is the major HAT enzyme responsible for acetylating H3K18 [32]. Using *in vitro* assembled chromatin we have shown that E1A 1-80 enhances p300 autoacetylation and inhibits p300-mediated H3K18 acetylation on chromatin [8]. Carey and colleagues have reported that during activated transcription, autoacetylated p300 dissociates from the promoter to facilitate the assembly of a pre-initiation complex [33]. However, when p300 is maintained at a hyper-acetylated state by knock-down of the histone deacetylase SIRT2, p300 transcriptional activity is diminished [34]. Since p300 autoacetylation is important for p300 activity [35], it appears that p300 is dynamically autoacetylated and deacetylated during transcription [34]. It is possible that E1A 1-80 helps maintain p300 at a hyper-acetylated state, thus inhibiting p300-mediated H3K18 acetylation on chromatin.

MYC transcription has been reported to be either down-or up-regulated by E1A 243R [36-39]. For example, in quiescent cells both E1A 243R and SV40 T antigen have been shown to induce MYC by targeting p300 [36, 40, 41]. In contrast, our analysis of E1A 1-80 expressed in cancer cells suggests that targeting of p300 by E1A 1-80 is correlated with inhibition of MYC promoter H3K18 acetylation and may contribute to MYC repression (Figures 3 and 6). Since E1A 243R interacts with proteins of multiple pathways of significance to cell proliferation [42], its function may be partly dependent on cell type, as we have demonstrated for the modulation of HER2 by E1A 1-80 (Figure 1). E1A 243R has been shown to interact with p400 and stabilize MYC, possibly as a way to enhance expression of MYC target genes [37]. However, a recent report suggests that stabilized MYC inhibits transcriptional elongation [43]. Thus, the correlation between MYC protein level and MYC transcriptional activity remains to be resolved. E1A 243R was also recently shown to associate with a MYC-TRRAP complex [44]. If E1A 1-80 also associates with the MYC-TRRAP complex, it may modulate the transcriptional activity of MYC protein and alter expression of MYC target genes. As a transcription factor, the MYC protein may recruit to its target promoters both p300/CBP and TRRAP-associated HAT enzymes including, for example, Tip60 and GCN5 [45-48]. Among these HAT enzymes, Tip60 preferentially acetylates H4K16 [18, 48]. Thus, the observation that E1A 1-80 targets TRRAP and inhibits H4K16 acetylation on the MYC promoter could be a potential model for E1A 1-80 regulation of MYC transcriptional functions (Figure 7).

In summary, the identification of MYC as a major regulatory gene targeted by E1A 1-80 for transcriptional repression and the involvement of both H3K18 acetylation and H4K16 acetylation in MYC repression is providing novel clues into the mechanism of MYC promoter regulation as well as E1A 1-80 transcriptional repression. Further understanding of the details of MYC regulation by E1A 1-80 may be valuable for cancer therapeutics targeting MYC.

## MATERIALS AND METHODS

### Cell culture

Human cancer cell lines SKBR3, MB231 (MB-MD231), MB468 (MB-MD468), MCF7, HeLa, HCT116, A549, and U2OS were cultured in DMEM supplemented with 4.5 g glucose/L, 5 mM glutamine, Pen/Strep, and 10% fetal bovine serum (Invitrogen) in a 37°C incubator with 5% CO_2_. Cells were passaged using Trypsin-EDTA (0.05% Trypsin, 0.25 mM EDTA, Invitrogen).

### Ad vector generation and purification

E1A 1-80 C+ Ad vector construction and preparation were as described previously [3]. Ad-E1A 1-80FH, Ad-∆2-11FH and Ad-∆26-35FH viral genomes were synthesized by GeneArt and cloned into the pAd-CMV-V5 vector (Invitrogen). These were transfected into 293A cells, and the resulted Ad vectors were amplified, purified by CsCl ultracentrifugation, and titered by plaque assay as described [49].

### Ad vector infection, RNA isolation, Reverse transcription (RT), and qPCR

For infection, cells were plated at a density of 3 × 10^5^ cells per well in 6-well plates (for RT-qPCR analysis) or 6 × 10^5^ cells per T25 flask (for RNA-seq analysis), and infected as described [3]. RNA was isolated with an RNA spin-column kit (5 PRIME) as recommended, except that cell lysates were passed through a QiaShredder spin column (Qiagen). RNA was reverse transcribed using Superscript II reverse transcriptase (Invitrogen) and (dT)18 primer. RT-qPCR primers (listed in Supplementary Table s2) were designed based on gene-specific primer sequences in PrimerBank [50, 51]. qPCR analysis was performed on an ABI 7500 with the SYBR Green JumpStart PCR kit (Sigma) with the inclusion of 5% DMSO, using the following program: one cycle of 95°C for 10 min pre-incubation, and 36 cycles of 95°C 10 s, 64°C 30 s. qPCR products were confirmed by melting curve analysis and gel electrophoresis.

### RNA-seq analysis

Triplicate samples of SKBR3 cells were infected with Ad-lacZ or Ad-E1A 1-80 C+, and RNA prepared as described above. RNA-seq analysis was performed with the assistance from the Washington University Genome Technology Access Center. PolyA^+^-RNAs from the samples were enriched prior to library construction.

### Chromatin immunoprecipitation (ChIP)

7.5 × 10^6^ SKBR3 cells were plated in T175 flasks, and infected with Ad vectors the next day. ChIP assays were performed using the MAGnify ChIP assay kit (Invitrogen) following manufacturer's recommendations. Formaldehyde-fixed cells were sonicated for a total of 2 min with alternate 15 s pulse and 15 s rest, at 10% amplitude using a Branson Digital Sonifier (model 450) with a microtip. For ChIP immunoprecipitation, 3 μg of antibody (or 3 μl of anti-serum) and 200 μl of 1:10 diluted chromatin were used. Input DNA controls represent 1% of the chromatin used for ChIP. Following ChIP, qPCR was performed under the same conditions as for RT-qPCR (primers listed in Supplementary Table s2).

### Antibodies

H4K16Ac: #SC-8662-R (Santa Cruz BioTech, Inc.), H3K18Ac: #07-354 (Millipore), RNA Pol II: #49-1033 (Invitrogen), E1A: rabbit polyclonal to E1A 1-80.

## SUPPLEMENTARY TABLES


